# Iron-Rich Conditions Induce OmpA and Virulence Changes of *Acinetobacter baumannii*

**DOI:** 10.3389/fmicb.2021.725194

**Published:** 2021-10-05

**Authors:** Hui Liu, Chun yuan Cao, Fu lan Qiu, Hao Nan Huang, Hongyan Xie, Renkang Dong, Yu Zhen Shi, Xiu Nian Hu

**Affiliations:** ^1^Department of Clinical Laboratory, Fujian Longyan First Hospital/Longyan First Affiliated Hospital of Fujian Medical University, Longyan, China; ^2^Department of Clinical Laboratory, Fujian Longyan Center for Disease Control and Prevention, Longyan, China; ^3^Intensive Care Unit, Fujian Longyan First Hospital/Longyan First Affiliated Hospital of Fujian Medical University, Longyan, China; ^4^Orthopaedic Surgery, Fujian Longyan First Hospital/Longyan First Affiliated Hospital of Fujian Medical University, Longyan, China

**Keywords:** *A. baumannii*, iron, OmpA protein, virulence, mice

## Abstract

**Background:** Iron ions affect the expression of outer membrane protein A (OmpA), a major pathogenic protein in *Acinetobacter baumannii.*

**Objective:** To analyze the effect of iron ions on the expression of the OmpA protein of *A. baumannii* and explore its association with the virulence of OmpA.

**Methods:** Site-directed mutagenesis was used to construct *ompA* gene deletion strains and gene repair strains. The OmpA protein expression of *A. baumannii* under culture with different contents of iron ions was detected. The virulence of *A. baumannii* with different OmpA protein expression levels were evaluated in macrophages and mice.

**Results:** OmpA protein levels of the three strains were enhanced under iron-rich conditions. They were reduced in the presence of the iron-chelating agent 2,2′-bipyridine. *A. baumannii* wild type and + *ompA* had a remarkable toxic effect on RAW246.7 macrophages (*P* < 0.05). In contrast, the Δ*ompA* had a significantly reduced toxic effect on RAW246.7 macrophages (*P* < 0.05). The levels of the inflammatory factors IL-1β, IL-6, IL-8, and TNFα in the mice spleen were significantly increased in the + *ompA* strain treatment group compared with the Δ*ompA* strain group (all *P* < 0.05). In addition, the levels were higher in the presence of iron ions than in the presence of the chelating agent.

**Conclusion:** Iron-rich conditions increase the OmpA protein expression of *A. baumannii*. Strains with high OmpA protein expression were more invasive, which may be a key determinant of *A. baumannii* infection and pathogenicity. Iron control strategies might be used for the management of *A. baumannii*.

## Introduction

*Acinetobacter baumannii* is a Gram-negative bacterium among the most important opportunistic pathogens for nosocomial infections, with strong acquired resistance and clonal spread capability (Peleg et al., 2008; Howard et al., 2012; Lee et al., 2017). Due to factors such as mechanical ventilation, use of broad-spectrum antibiotics, length of stay in ICU, and coma, critically ill patients with multidrug-resistant and pan drug-resistant *A. baumannii* infections often have a high mortality rate of 26.0–55.7%, with attributable mortality rates of 8.4–36.5% (Inchai et al., 2015; Uwingabiye et al., 2016; Xiao et al., 2017). According to the World Health Organization (WHO) report in 2017, carbapenem-resistant *A. baumannii* (CRAB) is currently the bacterium that poses the greatest threat to human health and for which new antibiotics are desperately needed (Willyard, 2017).

The outer membrane (OM) of Gram-negative bacteria is a unique architecture that acts as a defensive barrier to toxic molecules. It is composed of phospholipids, lipopolysaccharide (LPS), outer membrane β-barrel proteins (OMP), and lipoproteins (Dhilon and Patel, 2017; Choi and Lee, 2019). The outer membrane protein comprises heat-modifying protein, porin, and lipoprotein. They play an essential role in maintaining the bacterial structure, substance transport, cell surface recognition, signal transduction, and pathogenicity. However, they are also involved in physiological functions such as bacterial infection, adhesion, inflammation, activating the host to produce immune protection, and involvement in drug resistance (Nie et al., 2020; Viale and Evans, 2020). The outer membrane protein A (OmpA) is one of the components of OMPs of several Gram-negative bacilli. It is a key virulence factor that mediates bacterial biofilm formation, eukaryotic cell infection, antibiotic resistance, and immune regulation (Lee et al., 2017; Nie et al., 2020; Viale and Evans, 2020). An increase in the secretion of OmpA is independently associated with mortality in pneumonia and bacteremia caused by *A. baumannii* (Sanchez-Encinales et al., 2017). In addition, the mRNA expression level of *ompA* in *A. baumannii* is considered a rapid diagnostic index for antibiotics resistance (Martin-Pena et al., 2013). The OmpA is essential for *A. baumannii* to adhere to and invade epithelial cells and disseminate in blood and tissues (Gaddy et al., 2009; Parra-Millan et al., 2018). Therefore, OmpA is an attractive treatment target to fight *A. baumannii* infections (Nie et al., 2020).

Differential proteomics experiments revealed that the iron ion concentration affects the expression of the OmpA protein in *A. baumannii* (Nwugo et al., 2011). As an essential element and limited micronutrient for the survival of living organisms, iron ions are essential to maintain the activity of many intracellular enzymes (Abbaspour et al., 2014). In an aerobic environment, the iron available to cells is quite limited. After pathogenic bacterial infection, microorganisms must compete with the host to obtain iron (Zughaier and Cornelis, 2018). Mammals can restrict the utilization of iron by pathogenic bacteria through isolating intracellular iron and extracellular chelating iron using glycoproteins, transferrin, and lactoferrin. In contrast, bacteria have evolved a complex iron uptake system for competition with infected hosts (Dorsey et al., 2004).

Thus, traditional treatment strategies cannot effectively treat complicated infections caused by *A. baumannii*, especially CRAB and OmpA. As one of the most abundant protein in the OMPs of *A. baumannii* (Uppalapati et al., 2020), it is extensively involved in regulating physiological functions and deserves further research. Therefore, the present study aimed to analyze the effect of iron ions on the expression of the OmpA protein of *A. baumannii* and explore its association with the virulence of OmpA in mouse models.

## Materials and Methods

### Construction of the *ompA* Gene Deletion Strain (Δ*ompA*) of *A. baumannii*

The NCBI GenBank was used to retrieve the nucleotide sequence of *A. baumannii ompA* (GenBank: AY485227.1). Two pairs of specific PCR primers (P1A/P1B and P2A/P2B) were designed: *ompA*-P1A, 5′-GGAAAGTCTATCAAGTGTTTGTAGGATC CAAA-3′ (*Bam*HI); *ompA*-P1B, 5′-AACTTCTACTACAGGAG CAGCAGGCTCTCGAGAAA-3′ (*Xho*I); *ompA*-P2A, 5′-ATCA AGCCGTACGTATTATTAGGTGCTCGAGAAA-3′ (*Xho*I); and *ompA*-P2B, 5′-TTACTGTTCAAACT-3′ (*Xho*I). Using P1A/P1B and P2A/P2B as primers and the *A. baumannii* standard strain ATCC17978 [American Type Culture Collection (ATCC), United States]as a template, the left and right fragments of the *ompA* gene were amplified as the homology arms of homologous recombination. The pMD18-T (TaKaRa Bio, Japan) vector and the target fragment were ligated with DNA ligase (TaKaRa Bio, Japan) to transform DH5α (TaKaRa Bio, Japan) competent cells. Then, the positive plasmids were named p18T-*ompA*-P1 and p18T-*ompA*-P2 and verified by sequencing.

The recombinant plasmids p18T-*ompA*-P1 and p18T-*ompA*-P2 were extracted. *Bam*HI and *Xho*I was used for double digestion for the p18T-*ompA*-P1 recombinant plasmid. In contrast, *Xho*I and *Sph*I was used for double digestion for the p18T-*ompA*-P2 recombinant plasmid. The pMD18-T vector plasmid was subjected to double digestion with the *Bam*HI and *Sph*I. The target fragments were purified by a gel extraction kit.

Double digestion at 37°C for 3 h with *Sph*I and *Bam*HI was performed for the p18T-*ompA* recombinant plasmid and pDS132 (ATCC, United States) plasmid. The 6,000-bp band of pDS132 double digestion product and the 1,500-bp band of p18T-*ompA* double digestion product were extracted. The products extracted from the above two gel extraction kits were ligated overnight at 16°C. The ligation products were transformed into *E. coli* competent cells and the required pDS132-*ompA* recombinant plasmid were screened using LB (Sangon Biotech, China) plates containing Amp + (100 μg/mL). Then, a single colony was selected into the LB liquid medium containing the same concentration of Amp + and underwent shaking culture at 37°C. This was until the optical density (OD) value was about 1.0, which was measured at 600 nm. The positive recombinant plasmid after PCR and digestion identification was named pDS13-*ompA*.

For the screening of the Δ*ompA* of *A. baumannii*, it was constructed by conjugative transduction of a double-parent filter membrane (Griffiths et al., 2000). *E. coli* (pDS132-*ompA*) was used as the donor strain. The *A. baumannii* standard strain ATCC17978 was used as the recipient strain for conjugative transduction. The identification of the Δ*ompA* strain of *A. baumannii* was performed through a successful growth in Kanamycin (0.02 mg/mL) resistance selective medium and confirmed using western blot analysis and PCR identification.

### Construction of the *ompA* Gene Complementation Strain (*+ompA*) of *A. baumannii OmpA*

The primer sequences of the full-length *ompA* gene were designed and amplified (*ompA*-F1, 5′-GGAAAGTCTATCAA GTGTTTGTATG-3′; and *ompA*-R1: 5′-CGAGTCGCTTTTT TACTGTTCA-3′). Using the *A. baumannii* standard strain ATCC17978 as a template, the fragment size was about 1,235 bp. The PCR product of the amplified full-length *ompA* gene was subjected to gel electrophoresis. The DNA fragment was extracted and ligated to the pMD18-T vector and transformed into DH5α competent cells. The positive clone pT-*ompA* was selected and submitted to Genewiz Suzhou Biotechnology for sequencing. Double digestion at 37°C for 3 h with *Acc*I and *Bgl*II was performed for the pT-*ompA* recombinant plasmid and pWH1266 (ATCC, United States) vector plasmid.

The 7,000-bp band of the pWH1266 double digestion product and the 1,200-bp band of the pT-*ompA* double digestion product were extracted. The products extracted from the above two gel extraction kits were ligated overnight at 16°C, and the ligation products were transformed into Δ*ompA* competent cells. An LB plate containing Amp + (100 μg/mL) was used to screen the required pWH1266-*ompA* recombinant plasmid. A single colony was selected into the LB liquid medium containing the same concentration of Amp + and underwent shaking culture at 37°C until the OD value was about 1.0. After western blot and PCR confirmation, the Δ*ompA* + pWH1266-*ompA* strain (+ *ompA*) was obtained.

### Detection of Effects of Different Contents of Iron Ions on *ompA* Expression

The *A. baumannii* wild-type strain (*A. baumannii* standard strain ATCC17978), Δ*ompA* strain, and + *ompA* strain after activation overnight were transferred to the LB culture medium with 2mM FeSO_4_ added at 1:100. Then, transferred to the LB culture medium added with 350 μM (350 μmol/L) 2,2′-bipyridine solution for culture in the shaker at 37°C for 8–9 h and 3–4 h, respectively; then, the bacteria were collected. Western blot was used to detect the OmpA contents in the three groups.

### Dimethylthiazol-Diphenyltetrazolium Bromide (MTT) Colorimetry for Cytotoxicity Test

The concentration of RAW246.7 [National Collection of Authenticated Cell Cultures (NCTC), United Kingdom]macrophages was adjusted to 2 × 10^3^/mL with complete culture medium [10% fetal bovine serum (FBS) and 2% double-antibody were added to Dulbecco’s modified eagle medium (DMEM) purchased from Thermo Fisher Scientific, United States] and seeded in 96-well plates with 100 μL per well. The RAW246.7 macrophages were added with 8logCFU/mL of each *A. baumannii* strain. Normal saline was used as the control group. After 24 h of treatment, an MTT kit (cell proliferation kit I, Sigma-Aldrich, Germany)was used to detect cell viability. After adding MTT for 2 h, the Vario Skan Flash multi-functional microplate reader (Thermo Fisher Scientific, United States) was used to measure the optical density (OD) value of each well at 450 nm.

### Pathogenicity Test in Mice

Female nude mice (4–6-week-old) were obtained from Vital River Laboratory Animal Technology (Beijing, China) and kept in a specific pathogen-free environment at 25°C, 55% humidity, and 12-h light cycles with free access sterilized food and water. The experiments were approved by the animal ethics committee of the Ethics Committee of Longyan First Affiliated Hospital of Fujian Medical University, Longyan, Fujian, China (2017026 and 2020067). The mice were checked for their health status, and animal welfare supervision was provided by a certified veterinarian. All animal experiments were carried out in accordance with the Chinese governing laws on the use of medical laboratory animals (authorization no. 551998, 2013, by the Ministry of Health). Two *A. baumannii* wild-type strains, Δ*ompA* strain, and + *ompA* strain, were inoculated into LB culture medium. They were placed in a 37°C incubator for 20–24 h and then mixed with 10% (w/v) porcine mucoprotein at 1:1 to prepare a bacterial suspension. The bacterial content was determined by the spread plate method. The minimum lethal concentration and the maximum non-lethal concentration of the isolates for mice were first measured through preliminary testing. Then formal testing was carried out on this basis. First, 20 mice were randomly assigned to five groups (the experimental groups) and then administrated with 1 mL of normal saline and 1 mL of a bacterial suspension at an intraperitoneal injection dose of 5.7 × 10^8^ CFU/mL, 1.14 × 10^8^ CFU/mL, 2.28 × 10^7^ CFU/mL, 4.56 × 10^6^ CFU/mL, and 9.12 × 10^5^ CFU/mL. The blank control group was injected with 1 mL of normal saline. After 7 days of observation, the disease onset and death of mice and pathological necropsy changes were recorded. The modified Karber method (Rath et al., 2011) was used to calculate the LD50. The mice were sacrificed by cervical dislocation, and the spleens were immediately harvested and ground. Smears were prepared and stained with the Gram solution to observe the bacteria.

Spleen tissue homogenates (10%, wt/vol) were prepared with cold 1 × PBS (pH 7.2). TNF-α, IL-1β, IL-6, and IL-8 levels were measured in spleen tissues using mice enzyme-linked immunosorbent assay kits (Thermo Fisher Scientific, Waltham, MA, United States) according to the instructions.

### Western Blot Analysis

The strain of *A. baumannii* was lysed and collected with Bacterial Lysis Buffer (Cat# C500003, Sangon Biotech, China) supplemented with protease inhibitor cocktail (Cat# P8340, Roche). The lysis products were electrophoresed by sodium dodecyl sulfate-polyacrylamide gel electrophoresis (SDS-PAGE) and then transferred to polyvinylidene fluoride (PVDF) membranes. Incubation of the suggested primary antibodies *OmpA* (Abcam) and secondary antibodies (Abcam) was implemented in a 5% milk blocking reagent. Autoradiograms were quantified through densitometry controlled by GAPDH. The assay was repeated three times.

### qRT-PCR

Complete RNA was extracted from the strain of *A. baumannii* with TRIzol reagent (Cat# 15596018, Life Technologies) in keeping with the manufacturer’s instructions. Total RNA of 2 μg was reverse transcribed according to the protocol for the PrimeScript^®^ RT Master Mix Perfect Real-Time (Cat# RR047A, TaKaRa Bio, Japan). The qPCR reactions were implemented with SYBR Green Master Mix (Cat# RR820A, TaKaRa Bio, Japan) on a quantitative PCR instrument (Applied Biosystems 7900HT, Thermo Fisher Scientific).

### Statistical Analysis

All statistical analyses were performed using SPSS 22.0 (IBM, Armonk, NY, United States) and GraphPad Prism 5 (GraphPad Software Inc., San Diego, CA, United States). All continuous data are presented as means ± standard deviations and were analyzed using Student’s *t*-test or ANOVA with the LSD *post hoc* test. *P*-values < 0.05 were considered statistically significant.

## Results

### Detection of Effects of Iron Ion Contents on OmpA Expression

**FIGURE 1:**
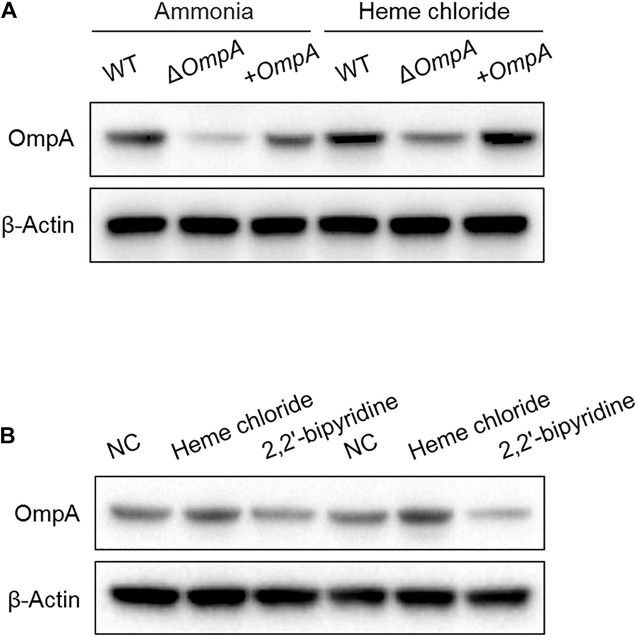
**(A)** Western blot analysis of the changes in OmpA protein expressions after treatment with hemin solution. **(B)** Detection of the effect of iron ion content on the OmpA expression. Two experiments were conducted with wild-type strains. *A. baumannii* standard strain ATCC17978). The *A. baumannii* wild-type strain was 5 × 10^7^ CFU/mL used.

The *A. baumannii* wild-type strain was 5 × 10^7^ CFU/mL used. The *ompA* expression of *A. baumannii* cultured in the LB culture medium added with 2mM FeSO_4_ was higher than that cultured after addition with 350 μM (350 μmol/L) of the iron-chelating agent 2,2′-bipyridine (Figure 1).

### Toxic Effect of Δ*ompA* Strain and + *ompA* Strain on Macrophages

**FIGURE 2:**
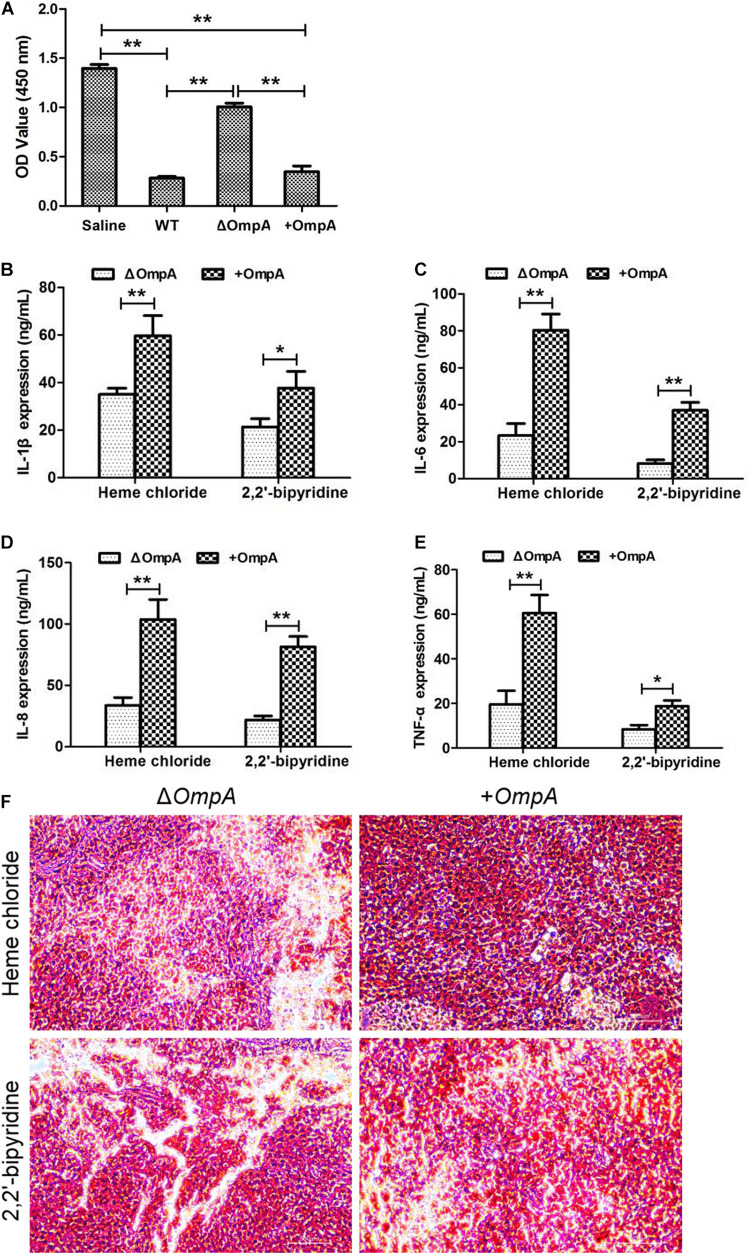
**(A)** Toxic effect of the Δ*ompA* and + *ompA* strains on macrophages. **(B–E)** The expression levels of inflammatory factors IL-1β, IL-6, IL-8, and TNFα in the mice spleen were significantly increased in the + *ompA* strain treatment group compared with the Δ*ompA* strain group. **(F)** The picture of spleen tissue grinding and Gram staining. Cells stained in red are bacteria. *P < 0.05 and **P < 0.01.

The results of the MTT test showed that the *A. baumannii* wild type and + *ompA* bacterial suspensions had a remarkable toxic effect on the RAW246.7 macrophages (*P* < *0.05*). On the other hand, the Δ*ompA* bacterial suspension had a significantly reduced toxic effect on the RAW246.7 macrophages (*P* < 0.05) (Figure 2A).

### Toxic Effect of Δ*ompA* Strain and *+ompA* Strain in Mice

The LD50 of the *A. baumannii* wild-type strain was 5 × 10^7^ CFU/mL (Table 1). The LD50 of the Δ*ompA* strain was > 5 × 10^8^ CFU/mL (Table 1). The LD50 of the + *ompA* strain was 5 × 10^7^–1 × 10^8^ CFU/mL (Table 1).

**TABLE 1:** The pathogenicity of *A. baumannii* wild, Δ*ompA, and* + *ompA* strain in mice.

Group	Dose/(CFU/mL)	No. of animals in each strain	Deaths and the mortality rate of *A. baumannii* wild		Deaths and the mortality rate of Δ *ompA*		Deaths and the mortality rate of + *ompA*	
			Deaths	Mortality rate	Deaths	Mortality rate	Deaths	Mortality rate
1	5 × 10^8^	4	4	100%	1	25%	3	75%
2	1 × 10^8^	4	4	100%	1	25%	2	50%
3	5 × 10^7^	4	2	50%	0	0%	2	50%
4	1 × 10^6^	4	1	25%	0	0	1	25%
5	5 × 10^5^	4	0	0	0	0	0	0
8	Normal saline	4	0	0	0	0	0	0

*CFU, colony-forming unit.*

The levels of the inflammatory factors IL-1β, IL-6, IL-8, and TNFα and the number of viable bacteria in the mice spleen were significantly increased in the + *ompA* strain treatment group compared with the Δ*ompA* strain group (all *P* < 0.05) (Figures 2B–F). In addition, the levels were higher in the presence of iron ions than in the presence of the chelating agent. The invasion of the spleen by the + *ompA* strain was higher than by the Δ*ompA* strain and higher in the presence of iron than in the presence of the chelating agent. The details of strain construction are provided in Supplementary Data and Supplementary Figure 1.

### Strain Construction

**FIGURE 3:**
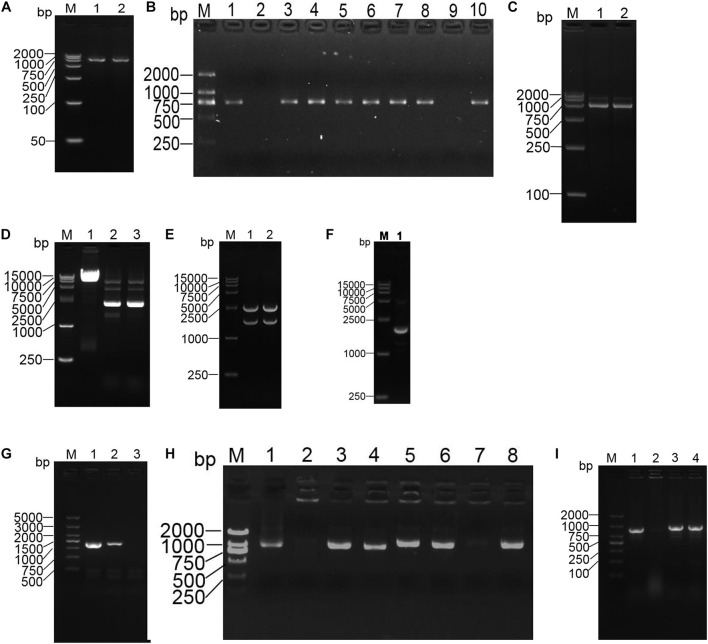
Construction of the *ompA* gene deletion and repair strains of the A. baumannii strains. **(A)** PCR amplification of fragments of the left and right homology arms of *ompA*. M: DL2000 marker; 1: the left arm of *ompA*; 2: the right arm of *ompA*. **(B)** Positive plasmid identified by PCR. M: DL2000 marker; 1Ű5: left arm identification; 6–10: right arm identification. **(C)** Identification of the recombinant plasmid p18T-*ompA* by digestion. M: DL2000 marker; 1: BamH I/Xho I for double digestion identification; 2: Xho I/Sph I for double digestion identification. **(D)** M: DL15000 marker; 1: pDS132 plasmid; 2Ű3: pDS132 plasmid identified with Sph I and BamH I double digestion fragments. **(E)** M: DL15000 marker; 1Ű2 p18T-*ompA* plasmid identified with Sph I and BamH I double digestion fragments. **(F)** Recombinant plasmid pDS132-*ompA* was identified by double digestion. M: DL15000 marker; 1: recombinant plasmid pDS132-*ompA* identified with double digestion fragments of Sph I and BamH I. **(G)** Identification of the *ompA* gene deletion strains. M: DL5000 marker; 1:16S rRNA amplified fragments; 2: amplified fragments of the *ompA* gene in the wild strain; 3: amplified fragments of the *ompA* gene deletion strain. **(H)** PCR identification: pWH1266-*ompA* positive plasmid M: DL2000 marker; 1–8: randomly selected pWH1266-*ompA* plasmid. **(I)** Identification of *ompA* gene deletion strains: M: DL2000 marker; 1: 16S rRNA amplified fragments; 2: amplified fragments of the *ompA* gene in the deletion strain; 3: amplified fragments of the *ompA* gene in the wild strain; 4: amplified fragments of the *ompA* gene in the repair strain.

The p18T-*ompA* recombinant plasmid was identified by double digestion with BamH I/XhoI and Xho I/Sph I, respectively. After digestion, an *ompA*-P1 fragment with a size of 753 bp was present and a size of 737 bp. The p18T-*ompA* recombinant plasmid and pDS132 plasmid were subjected to double digestion with Sph I and BamH I. After double digestion of pDS132, a 6000-bp band of the product was extracted. Similarly, after double digestion of p18T-*ompA*, a 1500-bp band of the product was extracted (see Figure 3).

## Discussion

*A. baumannii* plays an important role in nosocomial infections (Peleg et al., 2008; Howard et al., 2012; Lee et al., 2017; Wong et al., 2017; Harding et al., 2018). Iron ions affect the expression of OmpA, which is a major pathogenic protein in *A. baumannii.* This study aimed to analyze the effect of iron ions on the expression of the OmpA protein of *A. baumannii* and explore its association with the virulence of OmpA. The results suggest that iron-rich conditions increase the OmpA protein expression of *A. baumannii*. Strains with high OmpA protein expression were more invasive, which may be a key determinant of *A. baumannii* infection and pathogenicity. Iron control strategies might be used for the management of *A. baumannii*.

The OmpA is one of the most abundant protein in the outer membrane of *A. baumannii*. Its molecular weight is 38 kD (Maiti et al., 2011). The N-terminal domain is a β-barrel structure formed by 8-strand anti-parallel folding. The C-terminus is embedded in the peptidoglycan layer of the bacterial membrane and participates in the transmembrane transport of hydrophobic molecules (Jahangiri et al., 2017). AbOmpA amino acids isolated from various clinical strains are highly conserved (similarities > 89%), but they are heterologous for the human proteome (Jahangiri et al., 2017). When invasive infection occurs, *A. baumannii* first attaches to the host cell and then invades and transfers to the nucleus. After killing the host cell, it spreads in the bloodstream and tissues (Parra-Millan et al., 2018). In this process, OmpA is involved in mediating the adhesion and invasion of *A. baumannii* to epithelial cells (Schweppe et al., 2015). At the same time, OmpA contains abundant antigenic sites, which can induce host immunity in multiple ways and play an important role in *A. baumannii* control after colonization (McConnell et al., 2013). In order to further understand the function of OmpA, the present study analyzed the response of the *A. baumannii ompA* gene after inactivation and repair to host infection and pathogenicity. The present study showed that *ompA* deletion decreased the infectivity of *A. baumanniiin vitro* and *in vivo*. At the same time, repair increased infectivity, as supported by various studies (Martin-Pena et al., 2013; Lee et al., 2017; Sanchez-Encinales et al., 2017; Viale and Evans, 2020).

Still, this infectivity results from many complex factors, including iron (Gaddy et al., 2009), which is tightly controlled by hosts for aerobic energy production and to control infections (Abbaspour et al., 2014). Still, bacteria have evolved a complex iron uptake system to compete with the infected host (Dorsey et al., 2004). The free iron concentration in bacterial cells is mainly regulated by the iron uptake regulator (Fur). When the free iron concentration in the cell elevates, the Fur gene can bind to ferrous ions. Thereby inhibiting the genes coding of the iron uptake system and stimulating the genes coding the iron storage protein (Fontenot et al., 2020). In humans, transferrin prevents pathogens from contacting iron ions (Khan et al., 2018). The present study transferred various strains to LB culture medium added with 2 mM FeSO_4_. It then depleted free iron content in the cells using the iron-chelating agent 2,2′-bipyridine. Western blot suggested that the synthesis and expression of OmpA in the three groups were enhanced under iron-rich conditions. They were reduced in the presence of the iron-chelating agent, which confirms that the down-regulated phenotype of OmpA expression is caused, at least in part, by iron limitation.

In the *in vitro* pathogenicity study, the virulence test of *A. baumannii* on the RAW246.7 macrophages showed that the *A. baumannii* strain with OmpA was more invasive than that without OmpA, which had been further proved in the lethal infection experiment in mice. OmpA is needed for *A. baumannii* invasion (Gaddy et al., 2009), and mutations in *ompA* decrease the *A. baumannii* burden in mice (Choi et al., 2008). AbOmpA can directly cause the death of cells (Jin et al., 2011). AbOmpA also stimulates the innate immune response, as shown by higher inflammatory cytokines in the spleen of the mice (Akira and Hemmi, 2003; Trinchieri and Sher, 2007). In both cases, the virulence was higher in the presence of iron and lowered in the presence of the chelating agent. These results suggest that OmpA is a target for the management of *A. baumannii* infections and that iron-chelating agents could play a role in infection management, especially in the presence of pan drug-resistant *A. baumannii*. The actual methods that are being sought include OmpA -binding peptides, vaccines targeting OmpA epitopes, and monoclonal antibodies directly targeting OmpA (Nie et al., 2020). This study further suggests that iron chelation could also be used (Gentile et al., 2014).

This study has limitations. It only analyzed the effects of iron ions on the OmpA protein expression of *A. baumannii* and the pathogenicity of strains with different OmpA expressions. Whether iron-rich or iron-limited conditions promote the biofilm formation of *A. baumannii*, resistance to oxidative stress and other factors that affect the virulence phenotype remain to be studied.

In conclusion, the present study suggests that iron-rich conditions promote the OmpA protein expression of *A. baumannii*. The strains with high OmpA protein expression are more invasive, which may be a key determinant of *A. baumannii* infection and pathogenicity. Iron-dependent control strategies might be used to treat infectious diseases caused by pathogenic microorganisms.

## Data Availability Statement

The original contributions presented in the study are included in the article/Supplementary Material, further inquiries can be directed to the corresponding author/s.

## Ethics Statement

The animal study was reviewed and approved by the animal ethics committee of the Ethics Committee of Longyan First Affiliated Hospital of Fujian Medical University.

## Author Contributions

HL: manuscript preparation and funds collection. CYC: experiment involve. FLQ: literature search. HNH: statistical analysis. HYX: data collection. RKD: data interpretation. YZS: data interpretation. XNH: study design and data interpretation. All authors contributed to the article and approved the submitted version.

## Conflict of Interest

The authors declare that the research was conducted in the absence of any commercial or financial relationships that could be construed as a potential conflict of interest.

## Publisher’s Note

All claims expressed in this article are solely those of the authors and do not necessarily represent those of their affiliated organizations, or those of the publisher, the editors and the reviewers. Any product that may be evaluated in this article, or claim that may be made by its manufacturer, is not guaranteed or endorsed by the publisher.
