# Supplementary material for: Iron-Rich Conditions Induce OmpA and Virulence Changes of Acinetobacter baumannii
Source: Front Microbiol. 2021 Oct 5;12:725194. doi: 10.3389/fmicb.2021.725194 (PMC8525545; doi:10.3389/fmicb.2021.725194)

**Title: Iron-rich conditions induce *ompA* and virulence changes of *Acinetobacter baumannii***

**Supplemental data**

## Strain construction

The p18T-*ompA* recombinant plasmid was identified by double digestion with BamH I/XhoI and Xho I/Sph I, respectively. After digestion, an *ompA*-P1 fragment with a size of 753bp was present and a size of 737 bp. The p18T-*ompA* recombinant plasmid and pDS132 plasmid were subjected to double digestion with Sph I and BamH I. After double digestion of pDS132, a 6000-bp band of the product was extracted. Similarly, after double digestion of p18T-*ompA*, a 1500-bp band of the product was extracted (see Supplemental Figure S1).

**Supplemental Figure legend**

**Supplemental Figure 1.** Construction of the *ompA* gene deletion and repair strains of the *A. baumannii* strains. (A) PCR amplification of fragments of the left and right homology arms of *ompA*. M: DL2000 marker; 1: the left arm of *ompA*; 2: the right arm of *ompA*. (B)Positive plasmid identified by PCR. M: DL2000 marker; 1-5: left arm identification; 6-10: right arm identification. (C) Identification of the recombinant plasmid p18T-*ompA*by digestion. M: DL2000 marker; 1: BamH I/Xho I for double digestion identification; 2: Xho I/Sph I for double digestion identification. (D) M: DL15000 marker; 1: pDS132 plasmid; 2-3: pDS132 plasmid identified with Sph I and BamH I double digestion fragments. (E) M: DL15000 marker; 1-2 p18T-*ompA* plasmid identified with Sph I and BamH I double digestion fragments. (F) Recombinant plasmid pDS132-*ompA* was identified by double digestion. M: DL15000 marker; 1: recombinant plasmid pDS132-*ompA* identified with double digestion fragments of Sph I and BamH I. (G) Identification of the *ompA* gene deletion strains. M: DL5000 marker; 1:16S rRNA amplified fragments; 2: amplified fragments of the *ompA* gene in the wild strain; 3: amplified fragments of the *ompA* gene deletion strain. (H). PCR identification: pWH1266-*ompA* positive plasmid M: DL2000 marker; 1-8: randomly selected pWH1266-*ompA* plasmid. (I) Identification of *ompA* gene deletion strains: M: DL2000 marker; 1: 16S rRNA amplified fragments; 2: amplified fragments of the *ompA* gene in the deletion strain; 3: amplified fragments of the *ompA* gene in the wild strain; 4: amplified fragments of the *ompA* gene in the repair strain.

Supplemental Figure 1


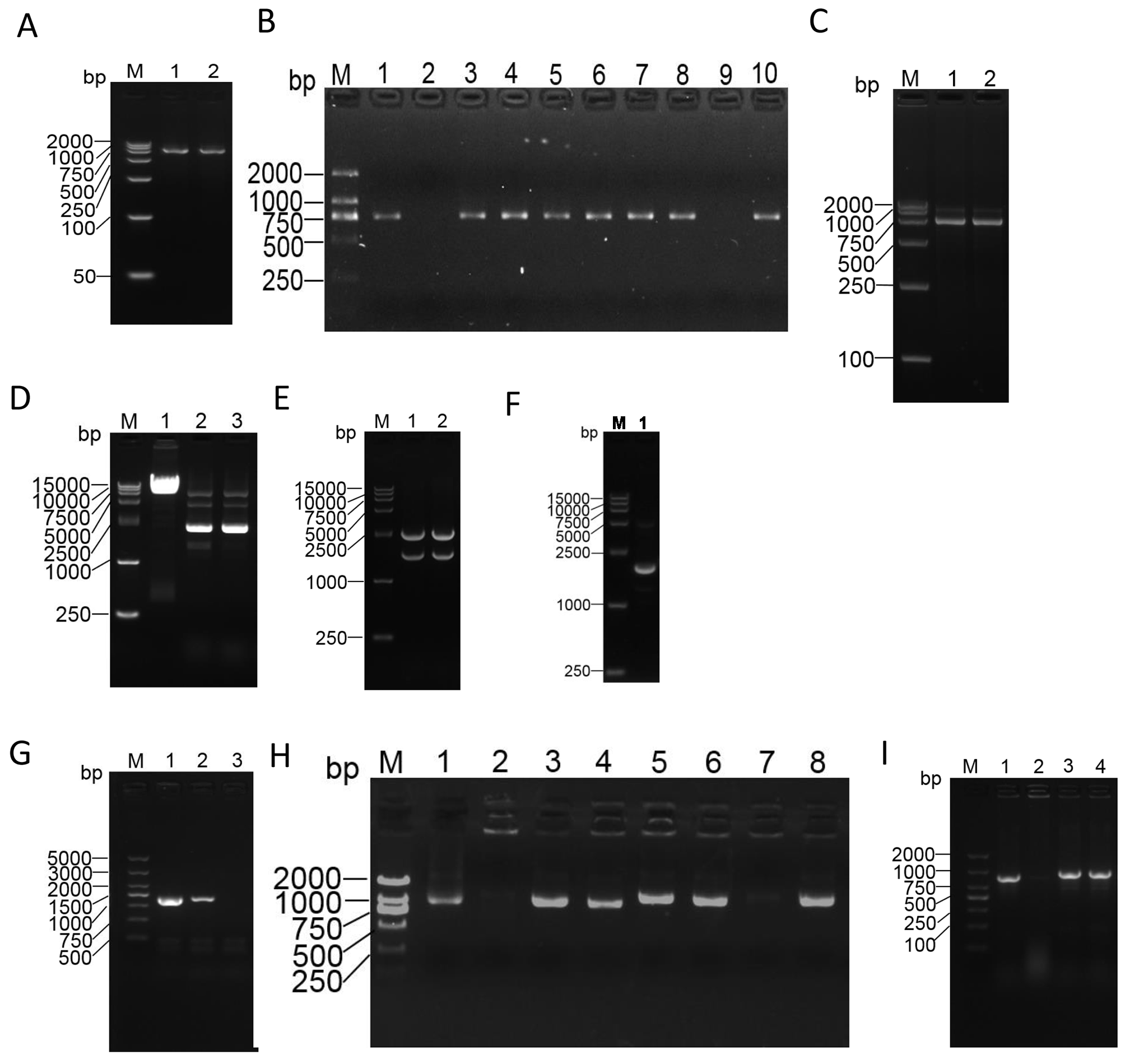

Supplement: Supplementary file 1 [file Data_Sheet_1.docx]
